# Low-dose insulin-like growth factor binding proteins 1 and 2 and angiopoietin-like protein 3 coordinately stimulate *ex vivo* expansion of human umbilical cord blood hematopoietic stem cells as assayed in NOD/SCID gamma null mice

**DOI:** 10.1186/scrt460

**Published:** 2014-05-30

**Authors:** Xiubo Fan, Florence PH Gay, Francesca WI Lim, Justina ML Ang, Pat PY Chu, Sudipto Bari, William YK Hwang

**Affiliations:** 1 Department of Clinical Research, Singapore General Hospital, 20 College Road, Academia Level 9, Discovery Tower, Singapore 169856, Singapore; 2 Cancer & Stem Cell Biology, Duke-NUS Graduate Medical School, College Road, Singapore 169857, Singapore; 3 Department of Hematology, Singapore General Hospital, 20 College Road, Academia Level 3, Singapore 169856, Singapore

## Abstract

**Introduction:**

Insulin-like growth factors (IGFs), IGF binding proteins (IGFBPs) and angiopoietin-like proteins (ANGPTLs) can enhance the *ex vivo* expansion of hematopoietic stem cells (HSCs) when used with a standard cytokine cocktail of stem cell factor (SCF), thrombopoietin (TPO) and FLT3 ligand (FL). In order to determine the optimal dose and combination of IGFs, IGFBPs and ANGPTLs, serial dilution and full permutation of IGFBP1, IGFBP2, IGF2 and ANGPTL3 were applied on a cryopreserved umbilical cord blood mononuclear cell (UCB-MNC) *ex vivo* expansion system.

**Methods:**

In this system, 4 × 10^5^ cells/ml of UCB-MNCs were inoculated in serum-free Stemspan® medium (Stemcell technologies, vancouver, BC, Canada) supplied with standard basal cytokine combination of 100 ng/ml SCF, 50 ng/ml FL and 100 ng/ml TPO and supported by a bone marrow mesenchymal stromal cell layer.

**Results:**

Paradoxically, experiment results showed that the highest expansion of CD34^+^CD38^−^CD90^+^ primitive progenitor was stimulated by cytokine combination of SCF + TPO + FL + IGFBP1 + IGFBP2 + ANGPTL3 at a low dose of 15 ng/ml IGFBP1 and 20 ng/ml IGFBP2 and ANGPTL3. This *ex vivo* expansion was further validated in 8-week-old to 10-week-old nonobese diabetic/severe combined immunodeficiency interleukin 2 gamma chain null (NOD/SCID-IL2Rγ^−/−^) mice. Limiting dilution assay showed excellent correlation between the HSC *ex vivo* surface marker of CD34^+^CD38^−^CD90^+^ and the *in vivo* competitive repopulating unit (CRU) functional assay.

**Conclusion:**

IGFBP1, IGFBP2, IGF2 and ANGPTL3 can stimulate the expansion of CD34^+^CD38^−^CD90^+^ primitive progenitor at low dose. The optimal combination comprises IGFBP1, IGFBP2 and ANGPTL3 together with the standard cytokine cocktail of SCF, FL and TPO. The CD34^+^CD38^−^CD90^+^ phenotype can serve as a surrogate *ex vivo* surface marker for HSCs due to consistency with the *in vivo* CRU functional assay.

## Introduction

*Ex vivo* expansion of umbilical cord blood (UCB) hematopoietic stem cells (HSCs) may overcome the obstacle of low cell dose for UCB transplantation in adults. Insulin-like growth factors (IGFs), insulin-like growth factor binding proteins (IGFBPs) and angiopoietin-like proteins (ANGPTLs) have been described previously to help enhance *ex vivo* expansion of HSCs when used with a standard cytokine cocktail of stem cell factor (SCF), thrombopoietin (TPO) and FLT3 ligand (FL) [1-10]. ANGPTLs and IGFBPs have also been demonstrated to enhance HSC *in vivo* migration and activity, supporting survival and replating capacity [11-14]. However, the optimal dose and combination of these novel cytokines have yet to be determined. Current doses of IGFBPs and ANGPTLs are in the range of 100 to 500 ng/ml. In terms of clinical application, these concentrations may not be optimal and would be costly. Hence, investigations into the optimal cytokine dose and combination of IGFs, IGFBPs and ANGPTLs are important.

In this study, serial dilution and full permutation were used to determine the optimal cytokine dose and combination for stimulation of *ex vivo* expansion of UCB-HSCs. This established cytokine dose and combination were then further validated in 8-week-old to 10-week-old nonobese diabetic/severe combined immunodeficiency interleukin 2 receptor gamma chain null (NOD/SCID-IL2Rγ^−/−^; NSG) mice.

## Methods

### Cell preparation

Cryopreserved UCB was obtained from Singapore Cord Blood Bank. Bone marrow (BM) was obtained from Singapore General Hospital with the donor’s informed consent. The use of UCB and BM was reviewed and approved by the Institutional Review Boards of National University of Singapore, Singapore General Hospital as well as the Singapore Cord Blood Bank Research Advisory Ethics Committee (for UCB). Cryopreserved UCB was processed in Singapore Cord Blood Bank following the standard volume reduction and red blood cell depletion method. The characteristics of the UCBs employed in this study are summarized in Table 1. BM-derived mesenchymal stromal cell culture was obtained as described in our previous publication [15,16].

**Table 1:** Cord blood unit phonotype information

**Unit used in experiment**	**CD34**^**+ **^**cells (%)**	**CD34**^**+**^**CD38**^**−**^**CD90**^**+ **^**cells (%)**
Figure 1	2.6	0.10
Figure 2	1.3	0.06
Figure 3	1.6	0.10
	0.9	0.05
	1.6	0.06
	0.8	0.03
	1.7	0.07
	1.5	0.06
Figures 4 and 5	1.6	0.08

### Cell culture

Cryopreserved UCB mononuclear cells (4 × 10^5^cells/ml) were suspended in serum-free Stemspan® medium (Stemcell technologies, vancouver, BC, Canada) supplied with a standard cytokine combination of 100 ng/ml SCF, 50 ng/ml FL and 100 ng/ml TPO (all three cytokines purchased from Peprotech, Rocky Hill, NJ, USA) and with individually varied doses and combinations of IGFBP1, IGFBP2, IGF2 and ANGPTL3 (these four cytokines purchased from R&D Systems, Minnneapolis, MN, USA). The cells were inoculated on a passage 3 to 5 BM-derived mesenchymal stromal cell layer and cultured in 37°C incubator for 12 days. The expanded cells were harvested at the end of 12 days and the adherent cord blood cells were detached after 1 minute of incubation at room temperature with 0.25% trypsin–ethylenediamine tetraacetic acid.

### Flow cytometric analysis

All data were acquired using the cytomics FC500 flow cytometer (Beckman Coulter, Inc., Miami, FL, USA) and 10,000 events per sample were collected. Cell viability (AnnV/7AAD), hematopoietic primitive progenitors (CD34/CD38/CD90) of unexpanded and *ex vivo* expanded UCB and human cell multi-lineage reconstitution (CD45, CD34, CD71, CD15/66b, CD3 and CD19/20) in mice were analyzed using the same method mentioned in our previously published paper [15,16].

### Methylcellulose colony assays

Quantification of granulocyte–macrophage colony-forming units was performed before and after expansion. The method was similar to our previous publication [15,16].

### NOD/SCID-IL2Rγ^−/−^mice transplantation

NSG mice were purchased from Jackson Laboratories (Klaine, USA) by SingHealth Experimental Medicine Centre and were maintained in the same facility. All animal experiments were performed under the approval of the SingHealth Institutional Animal Care and Use Committee. The unexpanded and expanded UCB with four different cytokine combinations, ‘SCF + TPO + FL’, ‘SCF + TPO + FL + IGFBP2’, ‘SCF + TPO + FL + IGFBP2 + IGF2 + ANGPTL3’ and ‘SCF + TPO + F + IGFBP1 + IGF2 + ANGPTL3’ at doses of 20 ng/ml IGFBP2 and ANGPTL3, 15 ng/ml IGFBP1 and 10 ng/ml IGF2, were injected intravenously via the tail vein into sublethally irradiated (240 cGy) 8-week-old to 10-week-old NSG mice. Acidified water and cyclosporine A were administered to NSG mice orally and by intraperitoneal injection for prophylaxis of bacterial and fungal infection and graft versus host disease.

### Harvesting of mouse bone marrow

At the end of the fourth month of transplantation, the mice were sacrificed using a carbon dioxide chamber. The femur and tibia were harvested and placed into cold RPMI medium (Invitrogen, Grand Island, NY, USA) immediately. Joint ends were cut and BM was flushed out with 10 ml of 2% fetal bovine serum–RPMI. Subsequently, contaminated red blood cells were lysed by ammonium chloride-based buffer before flow cytometric analysis.

### Statistical analysis

The results are expressed as mean ± standard deviation. The significance between two groups was determined using the two-sample independent *t* test. *P* < 0.05 was defined as significant. In terms of multiple comparisons, Bonferroni’s test was used to correct the *P* value for the *t* test. The processing and statistical analysis of the data was performed using OriginPro 7.5 software (OriginPro, Inc., Northampton, MA, USA).

## Results

### Low dose of IGFBP1, IGFBP2, IGF2 and ANGPTL3 is enough to boost the *ex vivo* expansion of CD34^+^CD38^−^CD90^+^ primitive progenitor cells

**Figure 1:**
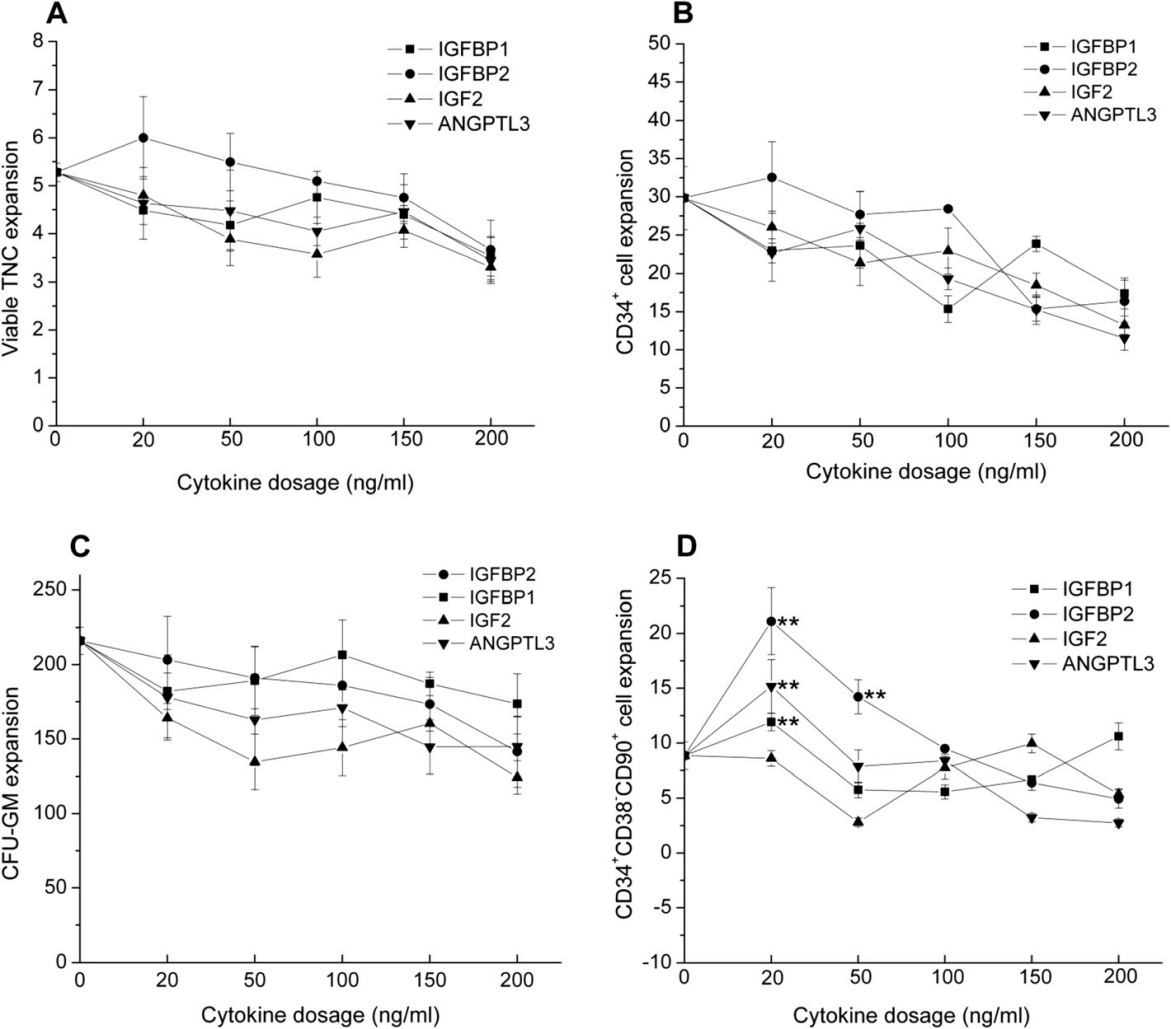
**IGFBP1, IGFBP2, IGF2 and ANGPTL3 stimulate the *ex vivo *expansion of CD34**^**+**^**CD38**^**−**^**CD90**^**+ **^**primitive progenitors at low dose.** Post-thaw umbilical cord blood cells (4 × 10^5^ cells/ml) were suspended in serum-free Stemspan® media (Stemcell technologies, vancouver, BC, Canada) supplied with different doses of indicated cytokines in the range 0 to 200 ng/ml and standard cytokine combinations of 100 ng/ml stem cell factor, 50 ng/ml FLT3 ligand and 100 ng/ml thrombopoietin, inoculated on a passage 3 to 5 bone marrow-derived mesenchymal stromal cell layer in a 24-well plate and cultured for 12 days. **(A)** Expansion of viable total nucleated cells (TNCs). **(B)** Expansion of CD34^+^ cells. **(C)** Expansion of granulocyte–macrophage colony-forming units (CFU-GM). **(D)** Expansion of CD34^+^CD38^−^CD90^+^ cells. Results expressed as mean ± standard deviation. For each novel cytokine, a *t* test was performed between the variant cytokine dosage group and baseline (standard cytokine combination). **P < 0.01. ANGPTL, angiopoietin-like protein; IGF, insulin-like growth factor; IGFBP, insulin-like growth factor binding protein.

**Figure 2:**
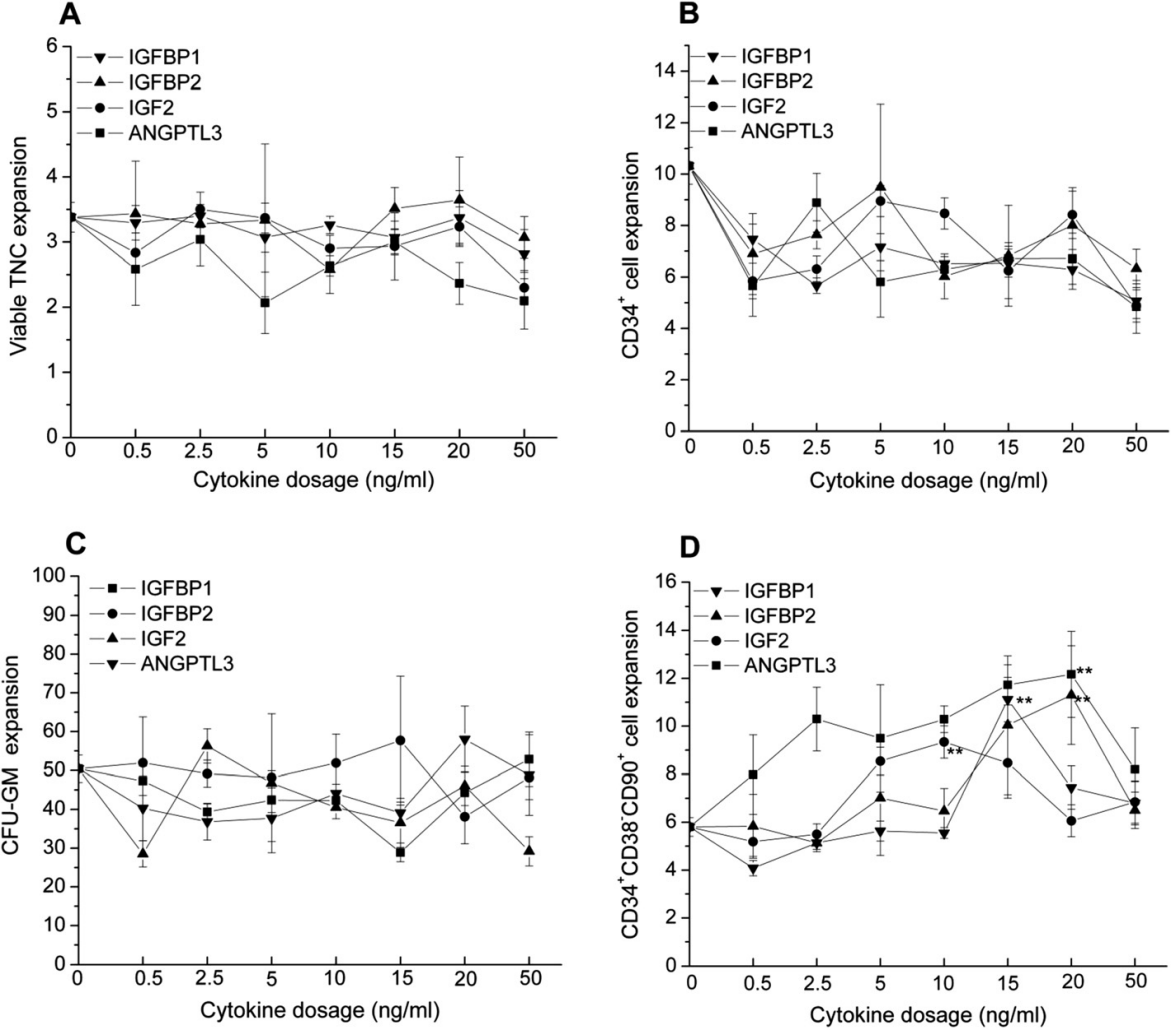
**Optimal dose of IGFBP1, IGFBP2, IGF2 and ANGPTL3 was established from 10 to 20 ng/ml.** Post-thaw umbilical cord blood cells (4 × 10^5^ cells/ml) were suspended in serum-free Stemspan® media (Stemcell technologies, vancouver, BC, Canada) supplied with different doses of indicated cytokines in the range 0 to 50 ng/ml and standard cytokine combinations of 100 ng/ml stem cell factor, 50 ng/ml FLT3 ligand and 100 ng/ml thrombopoietin, inoculated on a passage 3 to 5 bone marrow-derived mesenchymal stromal cell layer in a 24-well plate and cultured for 12 days. **(A)** Expansion of viable total nucleated cells (TNCs). **(B)** Expansion of CD34^+^ cells. **(C)** Expansion of granulocyte–macrophage colony-forming units (CFU-GM). **(D)** Expansion of CD34^+^CD38^−^CD90^+^ cells. Results expressed as mean ± standard deviation. For each novel cytokine, the *t* test was performed between the variant cytokine dosage group and baseline (standard cytokine combination). **P < 0.01. ANGPTL, angiopoietin-like protein; IGF, insulin-like growth factor; IGFBP, insulin-like growth factor binding protein.

When cryopreserved UCB mononuclear cells were cultured on a BM-derived mesenchymal stromal cell layer with the standard cytokine cocktail (100 ng/ml SCF, 50 ng/ml FL and 100 ng/ml TPO) plus individually varied doses of IGFBP1, IGFBP2, IGF2 and ANGPTL3, the highest expansion of CD34^+^CD38^−^CD90^+^ primitive progenitor appeared at a low dose of 20 ng/ml for IGFBP1, IGFBP2, IGF2 and ANGPTL3 (concentrations of 0, 20, 50, 100 and 200 ng/ml were studied) (Figure 1D). The expansion of total nucleated cells, CD34^+^ cells and granulocyte–macrophage colony-forming units decreased with increasing dose of cytokine (Figure 1A,B,C). Based on the above result, the range of cytokine dose was further narrowed to 0 to 50 ng/ml. The optimal cytokine dose was determined at 20 ng/ml IGFBP2 and ANGPTL3, 15 ng/ml IGFBP1 and 10 ng/ml IGF2 when maximal expansion of CD34^+^CD38^−^CD90^+^ primitive progenitor was reached, which was significantly higher than standard cytokine combination (*P* = 0.01) (Figure 2D). Similarly, these four novel cytokines did not contribute to the expansion of total nucleated cells, CD34^+^ cells and granulocyte–macrophage colony-forming units (Figure 2A,B,C).

### ‘SCF + TPO + FL + IGFBP1 + IGFBP2 + ANGPTL3’ is the optimal combination to enhance *ex vivo* expansion of CD34^+^CD38^−^CD90^+^ primitive progenitor cells

**Figure 3:**
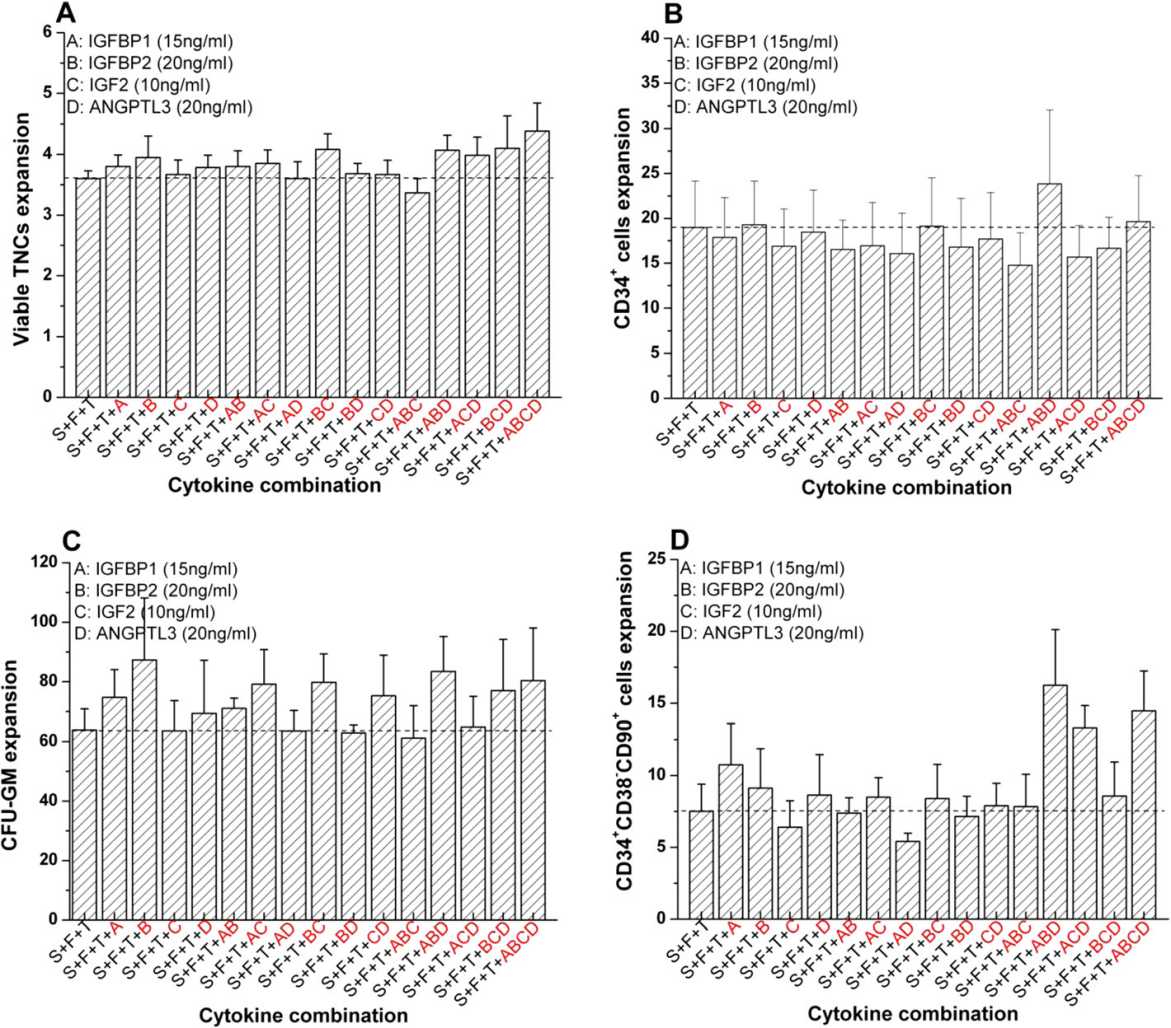
**‘SCF + TPO + FL + IGFBP1 + IGFBP2 + ANGPTL3’ is the optimal cytokine combination to enhance *ex vivo *expansion of CD34**^**+**^**CD38**^**−**^**CD90**^**+ **^**primitive progenitor cells.** Post-thaw umbilical cord blood cells (4 × 10^5^ cells/ml) were suspended in serum-free Stemspan® media (Stemcell technologies, vancouver, BC, Canada) supplied with different cytokine combinations with optimal doses and standard cytokine combinations of 100 ng/ml stem cell factor (S), 50 ng/ml FLT3 ligand (F) and 100 ng/ml thrombopoietin (T), inoculated on a passage 3 to 5 bone marrow-derived mesenchymal stromal cell layer in a 24-well plate and cultured for 12 days. Here, the optimal dose of IGFBP1 (A), IGFBP2 (B), IGF2 (C) and ANGPTL3 (D) was 15 ng/ml, 20 ng/ml, 10 ng/ml and 20 ng/ml respectively. **(A)** Expansion of viable total nucleated cells (TNCs). **(B)** Expansion of CD34^+^ cells. **(C)** Expansion of granulocyte–macrophage colony-forming units (CFU-GM). **(D)** Expansion of CD34^+^CD38^−^CD90^+^ cells. Results expressed as mean ± standard error (cord blood unit number, *n* = 6). ANGPTL, angiopoietin-like protein; IGF, insulin-like growth factor; IGFBP, insulin-like growth factor binding protein.

To determine the optimal cytokine combination, complete permutation was carried out after establishing the optimal dose of each cytokine. The combination ‘SCF + TPO + FL + IGFBP1 + IGFBP2 + ANGPTL3’ had superior expansion of CD34^+^CD38^−^CD90^+^ primitive progenitor (16.3 ± 3.9-fold versus 7.5 ± 1.9-fold with standard cytokine cocktail) compared with all other combinations (Figure 3D). Similarly, despite promoting expansion of CD34^+^CD38^−^CD90^+^ primitive cells, there was no further enhancement of expansion of total cells and general progenitors compared with control (Figure 3A,B,C), suggesting that this cytokine cocktail only enhanced the earlier progenitors. Hence, representative cytokine cocktails of ‘SCF + TPO + FL’, ‘SCF + TPO + FL + IGFBP2’, ‘SCF + TPO + FL + IGFBP2 + IGF2 + ANGPTL3’ and ‘SCF + TPO + FL + IGFBP1 + IGFBP2 + ANGPTL3’ were chosen for further validation in NSG mice.

### Equivalent human cell engraftment and multi-lineage reconstitution profile between unexpanded and expanded umbilical cord blood

**Figure 4:**
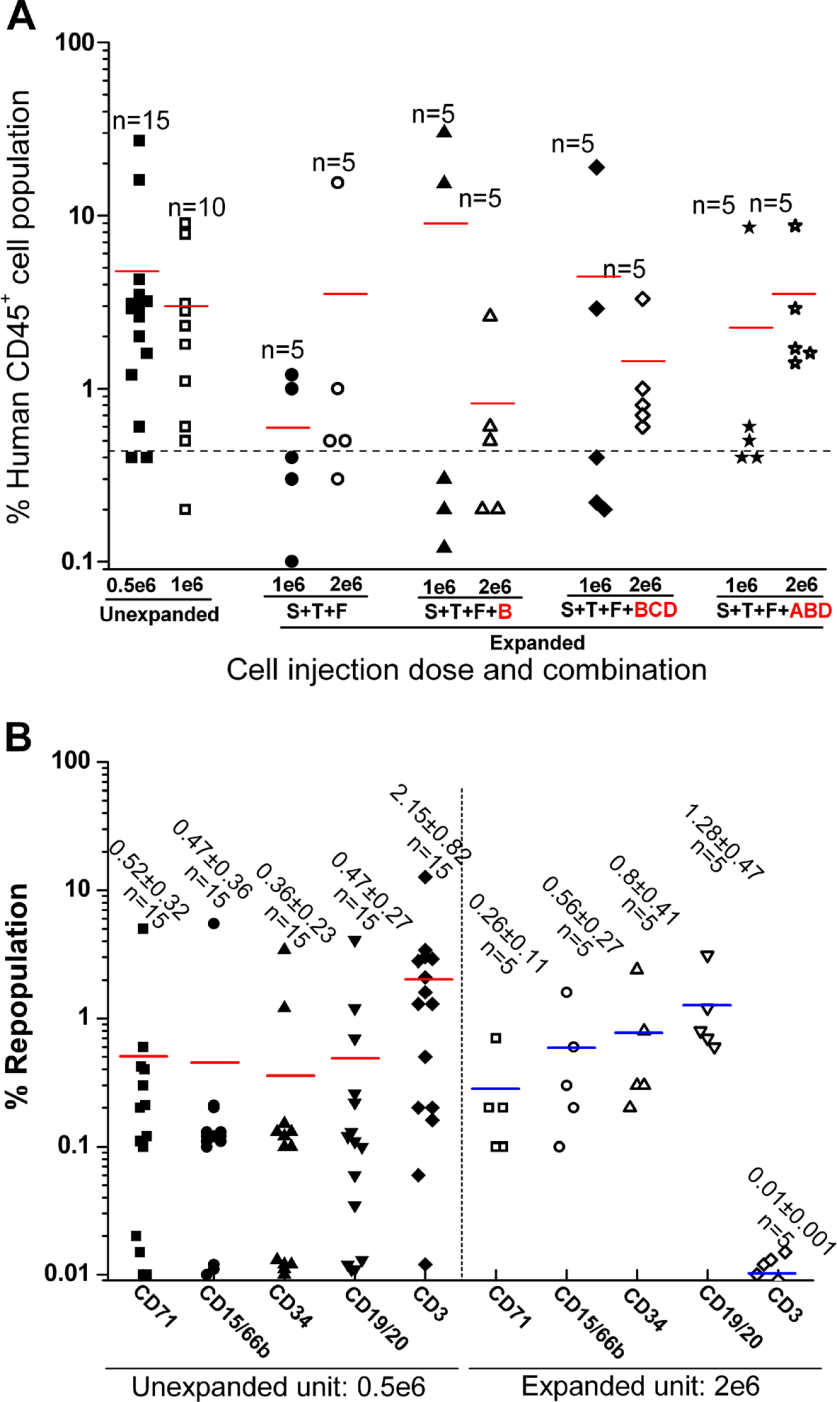
**Human cell engraftment and multi-lineage reconstitution profile. (A)** Amount of human chimerism in the bone marrow of NOD/SCID-IL2Rγ^−/−^mice that received a transplant of 5 × 10^5^ and 1 × 10^6^ unexpanded human mononuclear cord blood cells and 1 × 10^6^ and 2 × 10^6^ expanded progeny cells. Each symbol represents the engraftment of a single mouse that underwent transplantation assayed at 4 months post transplantation. **(B)** Summary of multi-lineage reconstitution from mice bone marrow transplanted 5 × 10^5^ unexpanded cells in lane 1 and transplanted 2 × 10^6^ expanded cells with the cytokine combination ‘S + F + T + ABD’ in lane 10 of **(A)**. S, stem cell factor; F, FLT3 ligand; T, thrombopoietin. A, IGFBP1; B, IGFBP2; C, IGF2; D, ANGPTL3. ANGPTL, angiopoietin-like protein; IGF, insulin-like growth factor; IGFBP, insulin-like growth factor binding protein.

NSG repopulation assays were performed to determine whether the *ex vivo* expanded cells were capable of long-term hematopoiesis. Thus, 5 × 10^5^ and 1 × 10^6^ unexpanded cells and 1 × 10^6^ and 2 × 10^6^ expanded cells with four cytokine combinations of ‘SCF + TPO + FL’, ‘SCF + TPO + FL + IGFBP2’, ‘SCF + TPO + FL + IGFBP2 + IGF2 + ANGPTL3’ and ‘SCF + TPO + FL + IGFBP1 + IGFBP2 + ANGPTL3’ were transplanted to irradiated NSG mice. The results did not demonstrate any significant difference in the human cell long-term engraftment when the mice received UCB expanded with cytokine combinations of ‘SCF + TPO + FL’, ‘SCF + TPO + FL + IGFBP2’, ‘SCF + TPO + FL + IGFBP2 + IGF2 + ANGPTL3’ and ‘SCF + TPO + FL + IGFBP1 + IGFBP2 + ANGPTL3’ compared with unexpanded UCB (*P* < 0.05; Figure 4A). This means that the stem cell repopulation properties were not lost during the *ex vivo* expansion. In addition, representative mice that received 2 × 10^6^ expanded cells cultured with the cytokine combination ‘SCF + FL + TPO + IGFBP1 + IGFBP2 + ANGPTL3’ displayed an equivalent multi-lineage differentiation capacity of primitive human cells (CD34^+^, 0.26 ± 0.11%), myeloid cells (CD45^+^CD71^+^, 0.2 ± 0.08%; CD15/66b^+^, 0.16 ± 0.07%), B-lymphoid cells (CD19/20^+^, 0.87 ± 0.56%) and T-lymphoid cells (CD3^+^, 0.01 ± 0.001%) to mice that received 0.5 × 10^6^ unexpanded cells of 0.36 ± 0.23%, 0.52 ± 0.32%, 0.47 ± 0.36%, 0.47 ± 0.27% and 2.15 ± 0.82% (*P* = 0.81, *P* = 0.58, *P* = 0.64, *P* = 0.50 and *P* = 0.15; Figure 4B). For T-lymphoid cell differentiation, even though there was no significant difference between expanded units and unexpanded units from statistical analysis, from direct observation we could say that the unexpanded unit was better than the expanded unit.

### Phenotypic marker of CD34^+^CD38^−^CD90^+^ can be used as a hematopoietic stem cell *ex vivo* detecting marker

**Figure 5:**
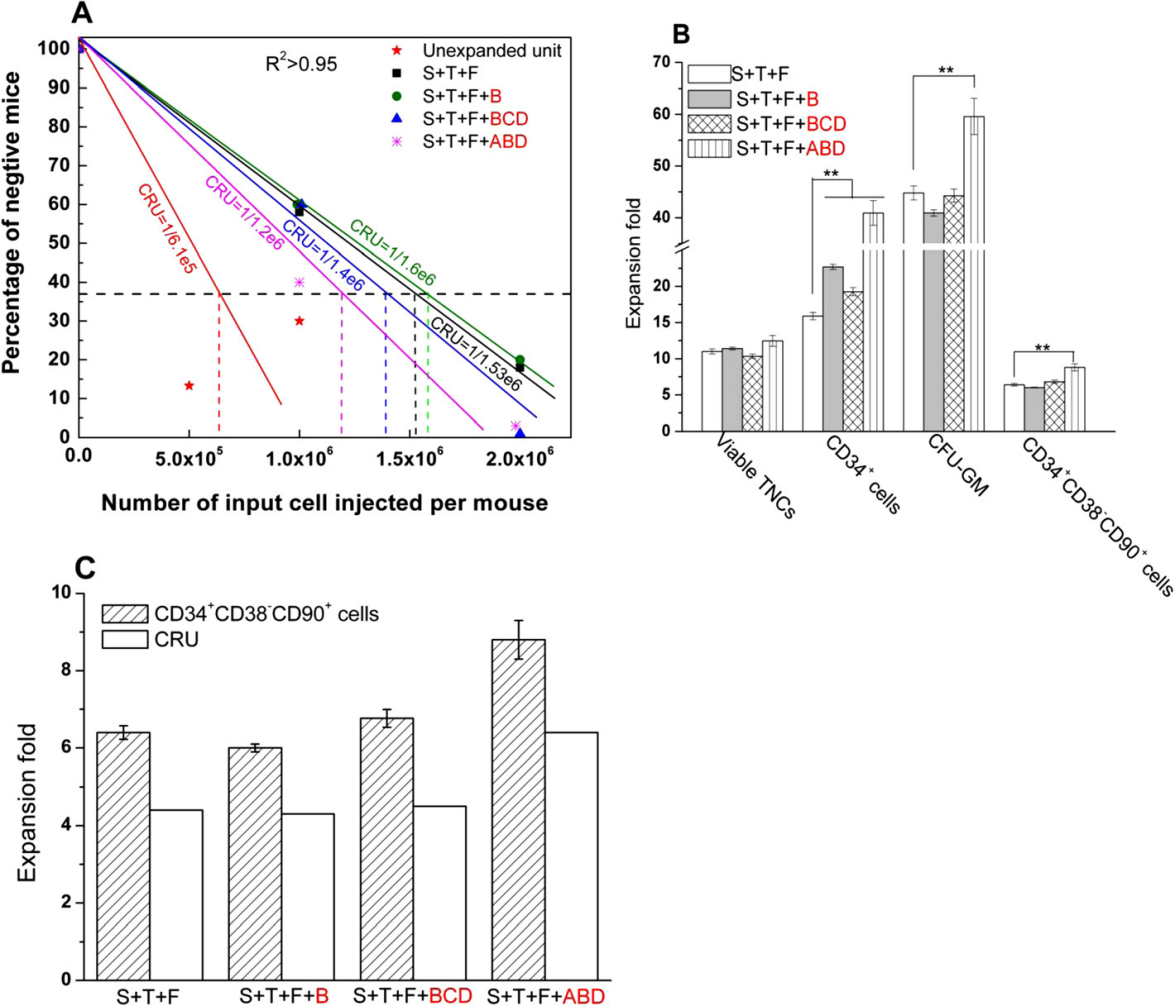
**Cytokine combination of ‘SCF + TPO + FL + IGFBP1 + IGFBP2 + ANGPTL3’ stimulates highest *ex vivo *expansion of primitive progenitors as assayed in NOD/SCID-IL2Rγ**^**−/−**^**mice. (A)** Limiting dilution assay for the unexpanded and expanded cells. Negative engraftment was defined by less than 0.5% of human CD45^+^ cell engraftment in mice bone marrow (*P* < 0.05). **(B)***Ex vivo* expansion of umbilical cord blood (UCB) cells. Post-thaw UCB cells (4 × 10^5^ cells/ml) were suspended in serum-free Stemspan® media (Stemcell technologies, vancouver, BC, Canada) supplied with cytokine combinations of ‘S + T + F’,’ S + T + F + B’,’ S + T + F + BCD’ and ‘S + T + F + ABD’ respectively, inoculated on a passage 3 to 5 bone marrow-derived mesenchymal stromal cell layer in a T_175_ flask and cultured for 12 days. Here, the concentration of stem cell factor (S), FLT3 ligand (F), thrombopoietin (T), IGFBP1 (A), IGFBP2 (B), IGF2 (C) and ANGPTL3 (D) was 100 ng/ml, 50 ng/ml, 100 ng/ml, 15 ng/ml, 20 ng/ml, 10 ng/ml and 20 ng/ml respectively. **(C)** Correlation of *in vivo* competitive repopulating unit (CRU) functional assay and *ex vivo* CD34^+^CD38^−^CD90^+^ cell surface marker. Results expressed as mean ± standard deviation. For multiple comparisons, Bonferroni’s test was used to correct the *P* value for the *t* test. *P* < 0.05 **→** *P*_corrected_ < 0.017 and *P* < 0.01 **→** *P*_corrected_ < 0.003 when *n* = 3. *P < 0.05, **P < 0.01. ANGPTL, angiopoietin-like protein; CFU-GM, granulocyte–macrophage colony-forming units; IGF, insulin-like growth factor; IGFBP, insulin-like growth factor binding protein; TNC, total nucleated cell.

After 4 months of transplantation, the limiting dilution assay showed that the competitive repopulating unit (CRU) was 1/1.53 × 10^6^, 1/1.6 × 10^6^, 1/1.4 × 10^6^ and 1/1.2 × 10^6^ in the expanded cells cultured with cytokine combinations of ‘SCF + TPO + FL’, ‘SCF + TPO + FL + IGFBP2’, ‘SCF + TPO + FL + IGFBP2 + IGF2 + ANGPTL3’ and ‘SCF + TPO + FL + IGFBP1 + IGFBP2 + ANGPTL3’ compared with 1/6.1 × 10^5^ in the unexpanded cells when *P* < 0.05 (Figure 5A). There was thus 4.4-fold, 4.3-fold, 4.5-fold and 6.4-fold *in vivo* expansion of long-term HSCs based on calculation according to the viable total nucleated cell expansion (Figure 5B, lane 1). There was 6.4 ± 0.2-fold, 6.0 ± 0.1-fold, 6.8 ± 0.2-fold and 8.8 ± 0.5-fold *ex vivo* expansion of CD34^+^CD38^−^CD90^+^ cells according to these four cytokine combinations (Figure 5B, lane 4). Strangely, there is always a close ratio of 69.8 ± 2.9% between *in vivo* expansion of the CRU and *ex vivo* expansion of CD34^+^CD38^−^CD90^+^ cells in different cytokine combinations (Figure 5C). The excellent correlation between the HSC *ex vivo* surface marker of CD34^+^CD38^−^CD90^+^ and the *in vivo* CRU functional assay indicate that phenotypic combination of CD34^+^CD38^−^CD90^+^ can be used as an HSC *ex vivo* detecting biomarker.

## Discussion

In this study, IGFBP1, IGFBP2, IGF2 and ANGPTL3 have been demonstrated to stimulate *ex vivo* expansion of CD34^+^CD38^−^CD90^+^ primitive progenitor at a low dose of 15 ng/ml IGFBP1, 10 ng/ml IGF2 and 20 ng/ml IGFBP2 and ANGPTL3. The optimal cytokine combination comprises IGFBP1, IGFBP2 and ANGPTL3 together with the standard cytokine cocktail of SCF, FL and TPO. In view of the excellent correlation between the HSC *ex vivo* surface marker of CD34^+^CD38^−^CD90^+^ and the *in vivo* CRU functional assay, the CD34^+^CD38^−^CD90^+^ phenotype can serve as an *ex vivo* surrogate surface marker for HSCs.

We showed that IGFBP1, IGFBP2, IGF2 and ANGPTL3 could stimulate the *ex vivo* expansion of HSCs or primitive progenitors rather than common progenitors and total cells, in agreement with recent reports [4,6,7,9,10]. However, this stimulation had a negative correlation to cytokine dose, where a high dose of cytokine induced low expansion of primitive progenitors. In fact, the optimal cytokine dose appeared at the lower range of 10 to 20 ng/ml. Compared with 100 to 500 ng/ml high-dose cytokine usage, this 10 to 20 ng/ml low dose will help to dramatically reduce the cost in clinical applications.

In addition, our data also showed that an expanded unit with the established optimal cytokine cocktail of ‘SCF + TPO + FL + IGFBP1 + IGFBP2 + ANGPTL3’ had the capacity of multi-lineage reconstitution except for T-lymphoid cells. Even though the statistical analysis showed that there was no significant difference on T-lymphoid cell differentiation between expanded units and unexpanded units, T-lymphoid reconstitution capacity from the unexpanded unit was better than from the expanded unit by direct observation. The explanation for this phenomenon could be due to the cytokine cocktail used in *ex vivo* expansion to drive cultured cells to expand and differentiate into the myeloid lineage rather than the T-lymphoid lineage (data not shown). This defect can be overcome by double cord blood transplantation with one expanded unit and one unexpanded unit as demonstrated in our previous publication [15,16].

From the limiting dilution assay, we know that only about 70% of expanded CD34^+^CD38^−^CD90^+^ primitive progenitors will reconstitute in NSG mice. What then happens to that 30% of expanded CD34^+^CD38^−^CD90^+^ primitive progenitors? We postulate two possibilities; the first is that 30% of expanded CD34^+^CD38^−^CD90^+^ primitive progenitors lose their *in vivo* reconstitution capacity during *ex vivo* expansion even though they express the biomarker phenotypically, while the second possibility could be that 70% of expanded CD34^+^CD38^−^CD90^+^ primitive progenitors possess self-renewal, pluripotency and long-term reconstitution capacity, whereas only 30% of them have short-term hematopoietic reconstitution capacity.

## Conclusions

IGFBP1, IGFBP2, IGF2 and ANGPTL3 can stimulate *ex vivo* expansion of CD34^+^CD38^−^CD90^+^ primitive progenitor at low dose with the optimal cytokine combination comprising IGFBP1, IGFBP2 and ANGPTL3 together with the standard cytokine cocktail of SCF, FL and TPO. The CD34^+^CD38^−^CD90^+^ phenotype can serve as a surrogate *ex vivo* surface marker for HSC detection due to consistency with the *in vivo* CRU functional assay.

## Abbreviations

ANGPTL: angiopoietin-like protein; BM: bone marrow; CRU: competitive repopulating unit; FL: FLT3 ligand; HSC: hematopoietic stem cell; IGFBP: insulin-like growth factor binding protein; IGF: insulin-like growth factor; NSG: nonobese diabetic/severe combined immunodeficiency interleukin 2 gamma chain null (NOD/SCID-IL2Rγ^−/−^); SCF: stem cell factor; TPO: thrombopoietin; UCB: umbilical cord blood.

## Competing interests

The authors declare that they have no competing interests.

## Authors’ contributions

XF was involved in conception of the study, experimental design, data interpretation, and drafting and revising the manuscript. FPHG and JMLA helped to carry out the colony-forming assay, flow cytometric assay and animal sample acquisition, and drafted the manuscript. FWIL provided suggestions and helped to revise the manuscript. PPYC and SB participated in the study design, data analysis and interpretation. WYKH had full access to all of the data in the study and takes responsibility for the integrity of the data and the accuracy of the data analysis. All authors read and approved the final manuscript.
